# Myocardial Performance Index Among Patients of Sickle Cell Disorder in Rural Teaching Hospital: A Case-Control Study

**DOI:** 10.7759/cureus.19175

**Published:** 2021-11-01

**Authors:** Anuj Chaturvedi, Sunil Kumar, Sourya Acharya, Shilpa A Gaidhane, Anil Wanjari, Dhruv Talwar

**Affiliations:** 1 Department of Medicine, Jawaharlal Nehru Medical College, Datta Meghe Institute of Medical Science (Deemed to be University), Wardha, IND; 2 Department of Medicine, Jawaharlal Nehru Medical College, Datta Meghe Institute of Medical Sciences (Deemed to be University), Wardha, IND; 3 School of Epidemiology and Public Health, Jawaharlal Nehru Medical College, Datta Meghe Institute of Medical Sciences (Deemed to be University), Wardha, IND

**Keywords:** cross sectional study, cardiac dysfunction, sickle cell crisis, myocardial performance index, sickle cell disease

## Abstract

Objective: To evaluate the myocardial performance index (MPI) in sickle cell disease (SCD) patients, and to compare MPI in healthy individuals.

Methods: In this case-controlled study, patients of SCD and age- and sex-matched controls were included. MPI was determined using echocardiographic Doppler-derived parameters. MPI according to disease type, sickle cell crisis, and in the presence of co-morbidities was compared.

Results: Between October 2018 and September 2020, we enrolled 100 SCD patients and 100 age and sex-matched controls. The MPI was significantly higher in SCD than in the control group (0.411±0.028 vs. 0.387±0.015, respectively; p<0.0001). There was no difference in MPI according to disease type as SS or AS (p=0.903). In patients with sickle cell crisis, mean MPI was significantly higher than those without sickle cell crisis (0.443±0.003 vs. 0.403±0.025, respectively; p<0.0001). In patients with comorbidities, mean MPI was significantly higher than those without any comorbidity (0.432±0.021 vs. 0.404±0.026, respectively; p<0.0001).

Conclusion: MPI can be a non-invasive tool for assessing subclinical cardiac dysfunction and should be considered for evaluating patients with SCD.

## Introduction

Sickle cell disease (SCD) is the second most common haemoglobinopathy [1]. Among countries in the low‐income category, up to 80% of SCD cases remain undiagnosed, and <50% among them survive beyond five years of age [2]. As per the estimates of the Anthropological Survey of India, the level of sickle cell train in some communities may be as high as 35% [3]. SCD is primarily observed in children and adolescents. However, with treatments that induce the protective fetal Hb and reduce the infections have improved the survival of SCD patients. With increasing survival into adulthood, the incidence of organ failure increases. The characteristic vaso-occlusive episodes in SCD may involve nearly every organ system [4,5]. Cardiovascular (CV) abnormalities are common and include pulmonary hypertension (PHT), dilatation of cardiac chambers, compensatory increase in left ventricular (LV) mass, and LV diastolic dysfunction. These result in a markedly low functional capacity, a high risk of severe multi-organ dysfunction, and sudden death [4,5]. In assessing LV systolic function, indices of the ejection phase are commonly measured. But, they have wide-ranging spectrum of results and apparent inconsistencies [6]. It is important to assess the cardiac function in these volume-overloaded patients using a load-independent measure [7]. In 1995, Tei and colleagues proposed a Doppler derived time interval index, i.e., Tei index or myocardial performance index (MPI). This index has good reproducibility and is not dependent on the LV geometry or the heart rate [8]. In SCD, cardiac involvement is not uncommon, but often remains underdiagnosed. Considering the high prevalence of SCD in India, and as the same is projected to rise in the future, we must have better understanding of cardiac involvement and the newer diagnostic dimensions in this disease. MPI may be a useful non-invasive and sensitive tool for SCD patients to assess the subclinical LV and RV dysfunction. There is a relative lack of studies evaluating the use of MPI in SCD in India, we conducted this study to evaluate the MPI in SCD patients, and to compare MPI in healthy individuals.

## Materials and methods

Design and participants

This was a case-control study at the medicine department of a rural teaching hospital in central India. Adults aged 18 years and above diagnosed or recently detected SCD attending to medicine OPD or admitted in the ward were included in the case group. Age- and sex-matched individuals without sickle cell anaemia were included in the control group. Patients with HbS/beta-thalassemia, haemorrhagic anemia, haemolytic anemia or aplastic anemia, ischemic heart disease or congenital heart disease or arrhythmia, acute myocardial infarction, rheumatic heart disease, angina pectoris were excluded. The institutional ethics committee approved the study with approval number Datta Meghe Institute of Medical Science (Deemed to be University)/Institutional ethics committee/2018-19/7554[DMIMS (DU)/IEC/2018-19/7554]. Informed consent from each participant was obtained before enrolment into the study.

Study procedures

Particulars such as name, age, sex, address, and telephone number were noted in a pre-structured proforma. The presence of comorbidities, personal habits, etc., were also noted. Hypertension was defined by assessing the blood pressure (BP) levels. Patients were labelled hypertensive if the BP was ≥140/90 mmHg or were taking drugs for the treatment of hypertension. Diabetes was diagnosed if the fasting blood glucose levels were >126 mg/dl or glycosylated Hb was 6.5% and above or the patient is taking antidiabetic medications to control glycemia. Body mass index was calculated using the standard formula as body weight (kg) divided by square of height (m). The classification of patients into the different categories of BMI was done as per the criteria for south Asians. SCD was detected by peripheral blood smear and confirmed by Hb electrophoresis. A sickle cell crisis was considered when a painful episode began suddenly. It is usually caused by when sickle-shaped red blood cells clump together and block small blood vessels. Sickle cell crisis is associated with mild to severe pain. Hb electrophoresis was performed using Uniclel DXH 800 machine. An automated cell counter machine of Horiba- Pentra XLR/ Horiba ABX Pentra XL80 was used for complete blood chemistry (CBC) assessment. Serum ferritin estimation was done using the VITROS® 5600 immunodiagnostic system employing the immuno-turbidimetry principle.

Measurement of MPI

Echocardiographic examinations of all participants were carried out using the Wipro ECHO machine (Wipro GE VIVID SR-460051 WXE, Ahmedabad, India). Using trans-mitral Doppler imaging, early diastolic peak flow velocity (E), late diastolic peak flow velocity (A), E/A ratio, E-wave deceleration time (DT), isovolumetric contraction time (IVCT), isovolumetric relaxation time (IVRT), and ejection time (ET) were determined. MPI was calculated by following the formula [8]: MPI = [IVRT + IVCT] / ET.

Statistical analysis

Data of participants were entered in Microsoft excel sheet and were analysed using the same. Categorical data were presented as frequency and percentages whereas continuous data as mean and standard deviation (SD). Chi-square test was used to determine statistical differences in categorical parameters whereas student t-test was used to determine statistical differences in continuous variables. P-value <0.05 was considered significant in all comparisons.

## Results

**Figure 1:**
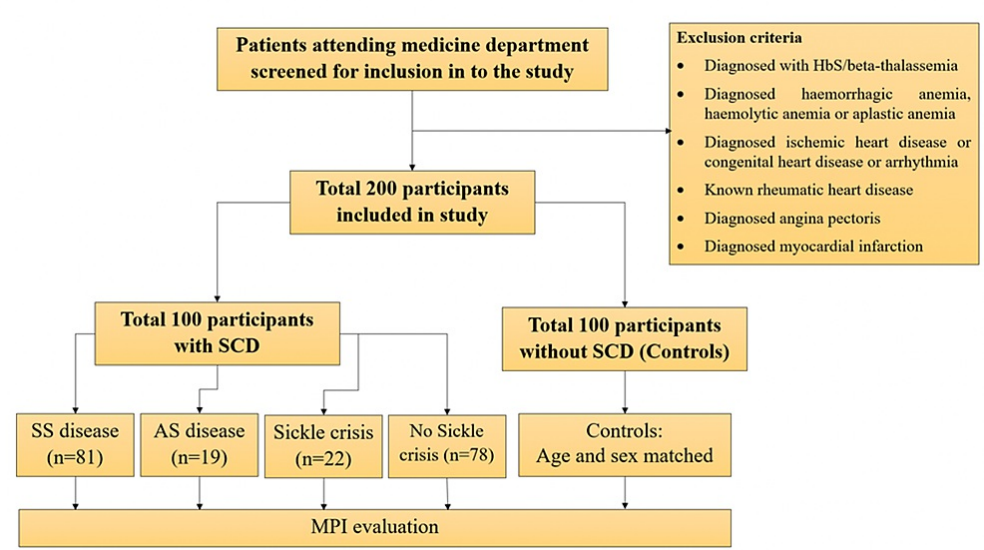
Study flow chart

Between October 2018 and September 2020, we enrolled 100 SCD patients and 100 age and sex-matched controls. Patients flow into the study is shown in Figure 1. In patients with SCD, SS-type was most common in 81% of cases whereas AS-type was seen in 19% of cases. Among SCD patients, 22% were in sickle cell crisis.

Baseline characteristics

Table 1 shows the baseline characteristics of study participants. The mean age of patients in the two groups was similar, i.e., 29.8±10.4 years. Diabetes was seen in 7% and 4% of patients from SCD and control groups, respectively; 21% of patients from the SCD group and 3% from the control group were alcoholic. The distribution was statistically significant (P<0.0001). Also, 24% and 3% of patients from SCD and control groups were smokers, respectively, and this distribution was statistically significant (p<0.0001). By level of Hb, 11% of patients from SCD had Hb <6 g/dl and 12% had Hb in between 6.1 and 7.9 g/dl whereas only one patient from the control group had Hb in this range. In the Hb range of 8 to 9.9 g/dL, there were 17% and 33% of patients from SCD and control groups, respectively.

**Table 1:** Baseline characteristics of cases and controls

Age (years)	Sickle cell disease (n=100)	Control (n=100)	P-value
Mean±SD	29.8±10.4	29.8±10.4	1.000 (NS)
Age groups			
≤25	40 (40.0)	40 (40.0)	1.000 (NS)
26–35	41 (41.0)	41 (41.0)	
36–45	11 (11.0)	11 (11.0)	
>45	8 (8.0)	8 (8.0)	
Gender			
Male	52 (52.0)	52 (52.0)	1.000 (NS)
Female	48 (48.0)	48 (48.0)	
Body mass index (kg/m^2^)			
Mean±SD	21.5±2.5	21.6±2.3	0.818 (NS)
Body mass index groups			
<18.0	0	0	0.938 (NS)
18.1–22.9	65 (65.0)	65 (65.0)	
23.0–24.9	30 (30.0)	31 (31.0)	
≥25.0	5 (5.0)	4 (4.0)	
Co-morbidities			
Hypertension	18 (18.0)	4 (4.0)	0.002 (S)
Diabetes	7 (7.0)	4 (4.0)	0.352 (NS)
Stroke	4 (4.0)	2 (2.0)	0.407 (NS)
≥2 comorbidities	26 (26.0)	4 (4.0)	<0.0001 (S)
Personal habits			
Alcoholic	21 (21.0)	3 (3.0)	<0.0001 (S)
Smoker	24 (24.0)	3 (3.0)	<0.0001 (S)
Haemoglobin (g/dl)			
Mean±SD	10.2±2.6	10.9±1.7	0.023 (S)
Hb groups			
<6.0	11 (11.0)	0	<0.0001 (S)
6.1–7.9	12 (12.0)	1 (1.0)	
8.0–9.9	17 (17.0)	33 (33.0)	
≥10	60 (60.0)	66 (66.0)	
Serum ferritin (ng/ml)			
Mean±SD	382.2±147.2	384.6±78.2	0.887 (NS)
≤300	35 (35.0)	25 (25.0)	0.123
>300	65 (65.0)	75 (75.0)	

The MPI was significantly higher in SCD than in the control group (0.411±0.028 vs. 0.387±0.015, respectively; p<0.0001). Table 2 represents the MPI distribution according to different subgroups. In AS-type SCD, the mean MPI was 0.411±0.028. In SS-type SCD, the mean MPI was 0.412±0.027. There was no significant difference in MPI value according to SCD type (p=0.903). In patients with sickle cell crisis, mean MPI was significantly higher than those without sickle cell crisis (0.443±0.003 vs. 0.403±0.025, respectively; p<0.0001). In patients with co-morbidities, mean MPI was significantly higher than those without any comorbidity (0.432±0.021 vs. 0.404±0.026, respectively; p<0.0001).

**Table 2:** Myocardial performance index comparison according to different subgroups

Subgroups	Myocardial performance index value	P-value
Sickle cell type		
AS Pattern	0.411±0.028	0.903 (NS)
SS Pattern	0.412±0.027	
Sickle cell crisis		
Present	0.443±0.003	<0.0001 (S)
Absent	0.403±0.025	
Any comorbidity		
Present (n=27)	0.432±0.021	<0.0001 (S)
Absent (n=73)	0.404±0.026	

## Discussion

In this case-control study, we included 100 patients with SCD and compared the observational parameters with 100 age and sex-matched control populations. We observed significantly higher MPI values in SCD than controls. Also, higher MPI was observed in those with crisis and had one or more comorbidity.

SCD usually presents in early childhood but children and adults may have similar symptoms. However, complications increase with age leading to early morbidity and mortality [9]. The mean age of patients in the two groups was similar, i.e., 29.8±10.4 years. A study by Chiadika et al. reported a similar age group with a mean age of patients with SCD was 33.1 ± 11.3 years [10]. The proportion of males in each group was similar, i.e., 52% and that of females was 48% in each group. A study from Dabirian et al. reported similar results with the proportion of males in the SCD and control group was 40.6% and 40.0% whereas that of females was 59.4% and 60.0%, respectively. The distribution was non-significant [11]. Though SCD in adults affected males and females with nearly equal frequency, evidence suggests males may have severe disease and proportionally over-represent among those required frequent hospitalizations in any index year [12]. Hypertension was seen in 18% of SCD patients and 4% of control patients. The distribution was statistically significant (p=0.002). Among adult patients with SCD, Chiadika et al. reported hypertension in 19% of patients [10]. A study from Benneh-Akwasi Kuma et al. reported a 19% prevalence of hypertension in adults with SCD [13]. HTN can contribute to increased mortality necessitating adequate control. The prevalence of stroke in our study was lower compared to observation from Chiadika et al. [10] and Shah et al. [14] who reported stroke in 10% and 9.8% of adult patients with SCD, respectively. Stroke is a devastating complication of SCD. Further, understanding the level of personal habits such as smoking is important in SCD patients as it is associated with a significant, clinically meaningful increased rate of ACS and pain events among adults with SCD [15].

The mean Hb of patients with SCD was significantly lower than the controls (10.2±2.6 vs. 10.9±1.7, respectively; p=0.023). Among adult patients with SCD, Chiadika et al. reported mean Hb levels of 9.0 ± 1.7 g/dl. Only 33% of patients had Hb 8 g/dl or lower [10]. Serum ferritin levels above 300 ng/ml were seen in 65% and 75% of patients from the two groups, respectively (p=0.123). Among adult patients with SCD, Chiadika et al. observed that 31% of patients had ferritin levels >1000 ng/ml [10]. Determining ferritin levels is of prognostic importance as high ferritin levels are found to be independently associated with one or more painful vaso-occlusive crises requiring an emergency visit or hospitalization for acute pain as well as with mortality [16].

The MPI was significantly higher in SCD than in the control group (0.411±0.028 vs. 0.387±0.015, respectively; p<0.0001). A similar study from Arslankoylu et al. observed that compared to the control group, children with SCD had significantly higher MPI of the left ventricle (0.319±0.109 vs. 0.175±0.053, p<0.0001) and right ventricle (0.223±0.058 vs. 0.171±0.047, p<0.0001) [17]. Another study from Caldas et al. reported similar observations with significantly higher MPI of left and right ventricle in patients with SCD than controls [18]. This indicates combined systolic and diastolic biventricular dysfunction in patients with SCD. We observed significantly higher MPI in patients with sickle cell crises and those with the presence of comorbidities. Sengupta et al. observed a significant reduction in peak LS in the subendocardial and subepicardial regions during the sickle cell crisis [19]. Identification of significantly higher MPI in SCD patients with comorbidities alarming as comorbidities also adversely affect cardiac function independent of SCD. 

Though our study provides one of the first evaluations of MPI in SCD in India, there are certain limitations. We excluded children from our study who are commonly affected with SCD. Comparison of MPI in children and adults would provide more information on the performance of this index in predicting ventricular dysfunction in two populations. We did not analyse the MPI by two genders which would have indicated whether the diastolic dysfunction differs by gender. The correlation of MPI with other markers of diastolic dysfunction would have helped extend its clinical importance in the management of SCD.

## Conclusions

SCD is associated with a variety of complications of the CV system ranging from elevated pulmonary artery systolic pressure, PHT, dysrhythmia, cardiomyopathies, and sudden cardiac death. The development of LV systolic and diastolic dysfunction can be associated with increased mortality in SCD patients. Early identification of such dysfunction can be helpful to determine the management approach in SCD patients. MPI has been identified as a non-invasive tool for assessing the subclinical cardiac LV and RV dysfunction in patients with SCD. We observed higher levels of MPI in SCD patients than control population indicating the presence of ventricular dysfunction in adults with SCD. Therefore, MPI should be considered for evaluating the RV and LV dysfunction in patients with SCD. If the Rural Hospitals of India become well equipped with echography machines, it would enable the root care primary centres to detect ventricular dysfunction through the MPI in the sickle cell population thus reducing morbidity and mortality.
